# Neglect of a Neglected Disease in Italy: The Challenge of Access-to-Care for Chagas Disease in Bergamo Area

**DOI:** 10.1371/journal.pntd.0004103

**Published:** 2015-09-25

**Authors:** Ernestina Carla Repetto, Rony Zachariah, Ajay Kumar, Andrea Angheben, Federico Gobbi, Mariella Anselmi, Ahmad Al Rousan, Carlota Torrico, Rosa Ruiz, Gabriel Ledezma, Maria Chiara Buoninsegna, Mohammed Khogali, Rafael Van den Bergh, Gianfranco De Maio, Ada Maristella Egidi, Barbara Maccagno, Silvia Garelli

**Affiliations:** 1 Médecins Sans Frontières, Operational Centre Brussels, MSF-Belgium, Brussels, Belgium; 2 Médecins Sans Frontières, Medical Department, Operational Centre Brussels, MSF-Luxembourg, Luxembourg, Luxembourg; 3 International Union Against Tuberculosis and Lung Disease, South-East Asia Regional Office, New Delhi, India; 4 Centre of Tropical Diseases, Sacro Cuore Hospital, Negrar-Verona, Italy; 5 COHEMI Project, Milan, Italy; 6 Centre of Tropical Diseases and Community Epidemiology (CECOMET) Esmeraldas, Ecuador; 7 OIKOS Onlus, Bergamo, Italy; 8 Médecins Sans Frontières, Medical Department, Operational Centre Brussels, MSF-Italy, Rome, Italy; Federal University of São Paulo, BRAZIL

## Abstract

**Objectives:**

Chagas disease (CD) represents a growing problem in Europe; Italy is one of the most affected countries but there is no national framework for CD and access-to-care is challenging. In 2012 Médecins Sans Frontières (MSF) started an intervention in Bergamo province, where many people of Latin American origin (PLAO) are resident. A new model-of-care for CD, initiated by Centre for Tropical Diseases of Sacro Cuore Hospital, Negrar (CTD), the NGO OIKOS and the Bolivian community since 2009 in the same area, was endorsed. Hereby, we aim to describe the prevalence of CD and the treatment management outcomes among PLAO screened from 1^st^ June 2012 to 30^th^ June 2013.

**Methods:**

Retrospective cohort study using routine program data. Screening sessions were done in Bergamo at OIKOS outpatient service and serological confirmation, staging and treatment for CD was offered at the CTD. MSF provided health education on CD, awareness generation prior to screening days, pre-test and post-test counselling through cultural mediators of Latin American origin.

**Results:**

Of 1305 PLAO screened, 223(17%) had CD. Among 210 patients eligible for treatment, 102(49%) were lost-to-follow-up before treatment. The median delay from diagnosis to treatment was 4 months (range 0.7–16.6 months). Among 108 started on treatment, 63(58%) completed treatment, 36(33%) interrupted treatment, (33 for drug side-effects, two for patients decision and one due to pregnancy), 6(6%) were lost-to-follow-up and 3(3%) were on treatment at study censuring.

**Conclusion:**

In this first study focusing on process of care for CD in Italy, less than 30% of patients completed treatment with drop-outs along the cascade of care. There is an urgent need to involve affected communities and local regional health authorities to take part to this model-of-care, adapting it to the local epidemiology. The Italian health authorities should take steps in advocating for a change in the current paradigm.

## Author Summary

Chagas disease is a neglected disease which, when left untreated, leads to severe cardiac and or digestive problems in 40% of the affected individuals. Globally, less than 1% of affected people have access to care. While mainly documented in Latin America, Chagas disease is not limited to this region due to population movements; however, awareness and provision of care are poor globally. Because of the relatively high levels of Chagas disease reported in Italy and the lack of information on programmatic management of the disease outside of Latin America, we aimed to analyse the outcome of an integrated program of health education, free universal screening, and free access to care, managed in collaboration between the NGOs Médecins Sans Frontieres and OIKOS, and the Centre of Tropical Diseases of Negrar Hospital in Verona province, with the active participation and empowerment of the local communities. Our results show that the prevalence of Chagas disease among people of Latin American origin in Bergamo province was high and that, despite an increased access to diagnosis and treatment in the program, many people did not complete the full course of treatment. More efforts should be done by the Italian authorities to facilitate access to care.

## Introduction

Chagas disease (CD), also known as American Trypanosomiasis, is caused by the protozoan parasite *Trypanosoma cruzi*. According to the World Health Organization (WHO) there are about 8 million [1] estimated cases of CD worldwide, and about 11,000 attributed annual global deaths [2]. The number is probably underestimated, since recent projections do assign only to North America (comprising Mexico) between 1.3 and 7 million cases [3].

The disease is mainly transmitted to animals and people through the faeces of insect vectors—the blood sucking triatominae bugs known as the“kissing bugs” [4]. Other methods of transmission include blood transfusions, organ transplantation, ingestion of food contaminated with bug stool and mother-to-child transmission.

Following infection, whatever the modality, there is an *acute phase* which often goes unnoticed or is associated with mild symptoms such as fever, fatigue, headache and occasionally swellings at the site of insect bite (Chagomas) or generalized oedema. If patient is untreated or inadequately treated, a *chronic phase* follows during which the infection may remain unnoticed for several decades or even for life. However, up to 40% of patients may develop serious, life-threatening cardiovascular and/or gastrointestinal or neurological complications that may even result in sudden death [5]. Given the difficulty in predicting who will develop complications, all diagnosed individuals should be offered treatment in order to reduce disease transmission risk [6] and evolution [7]. However, only 1% of the globally infected population have access to diagnosis and treatment (a 99% access gap)[5, 8, 28].

This, along with the fact that the available drugs are over half a century old and have a considerable level of toxicity, there is no rapid test for cure for chronic infections, and there has been limited research on new diagnostics and drugs, contributes to make CD a “neglected disease”. In order to overcome the global impact of neglected diseases including CD, the WHO in 2012, launched a roadmap for its elimination: among others [9] the two pillars of the strategy include a) providing treatment and care for patients and b) interrupting disease transmission by 2020.

The disease, which was once entirely confined to Latin America, has now spread to other continents, due to population movements. In Europe, there are an estimated 100,000 infected people [10] and Italy is one of the most affected countries with about 12,000 infected persons[11]. Limited published evidence on prevalence of CD among Bolivians in Europe revealed a prevalence ranging from 6.8 to 25%, the lowest being the Netherland and the highest in Spain[12,13]. Although there are no Italian prevalence data and hospital-based studies may be compromised by selection bias which may overestimate infection rates, recent surveillance reports from a selected tropical diseases center indicate that up to 11% of people of Latin American Origin (PLAO) in Italy have CD, with the highest level (30%) seen among people from Bolivia[11].Further, there is no national framework for the control and management of CD and treatment is only available in a few tertiary hospitals.

In order to bridge the existing access gap in Italy, Médecins Sans Frontières (MSF) in 2012 started to empower an unique model-of-care for Chagas disease, ongoing since 2009 for PLAO residing in the province of Bergamo, an area with the estimated major concentration of CD affected people in Italy.

A PubMed search revealed that there is only one paper from Spain on treatment management outcomes [14] and no papers documenting the journey from screening to treatment including the pre-treatment phase where losses to follow up may occur. Such information would be highly beneficial to inform control efforts.

Among PLAO screened for CD in Bergamo, we thus aimed to describe the prevalence of the disease and the management outcomes. The specific objectives were to report on a) the numbers screened and diagnosed with Chagas b) the numbers eligible and initiated on treatment c) the time delay between diagnosis and treatment initiation d) the end of treatment management outcomes and e) the drug related adverse effects.

## Methods

### Study design

Retrospective cohort study using routine program data.

### Study setting

The study was conducted in the OIKOS out-patient clinic located in Bergamo town, Northern Italy. Bergamo town is the capital of Bergamo province, one of the largest provinces in the Lombardia region, and has a population of about 115,000 inhabitants. The Bergamo province hosts about a million inhabitants including about 120,800 migrants[15]. Migrants come from various countries including about 11,000 from Latin America countries, of whom over 50% were from Bolivia[16].

### The model-of-care for Chagas disease in Bergamo

#### Collaborative partnership

This project was conducted in collaboration with the Centre of Tropical Diseases (CTD) of Sacro Cuore Hospital of Negrar town, Northern Italy (province of Verona, Veneto region). This is located at about 130 km from Bergamo and is one of the few hospitals specialized in managing CD in Italy. The other partner was a non-governmental organization (NGO)-OIKOS Onlus, which manages an outpatient service in Bergamo dedicated to health care assistance for undocumented migrants.

#### Community awareness raising and patient screening

Since 2009 health education on CD was promoted by the Bergamo Bolivian community and CTD and periodic screening sessions were on-going in the study area, mainly based at OIKOS ambulatories and on a voluntary basis. Screening sessions took place around 3–5 times yearly and people found positive for *T*. *cruzi* antibodies were informed and invited to go to CTD for staging and treatment. The program permitted to offer diagnosis to around 300–400 persons and to treat around 60 patients, yearly. MSF enforced this program providing regular health education on CD, awareness generation prior to screening days, pre-test and post-test counselling through cultural mediators of Latin American origin, specially trained for the purpose. Screening days were organized at the OIKOS outpatient service on monthly basis in order to assure a better coverage of the estimated population at risk (around 10000 people). All PLAO attending the outpatient clinic during those days were systematically interviewed by a physician or a nurse, in order to assess risk factors for CD (country of origin, home country surroundings, type of home country housing, blood transfusion, etc.). Following informed consent, blood samples were collected from all patients attending the clinic and transported from the OIKOS outpatient service to the CTD laboratory in Negrar on the same day by a courier service. Diagnostic testing was done using two serological ELISA essays (Chagas III, BiosChile and BioELISA Chagas, Biokit) both of which have sensitivity and specificity >99%[17]. The two tests based on different antigens were used in parallel to increase the accuracy of the diagnosis [18]. A case was considered positive if both tests were positive, while discordance was termed as indeterminate case. Indeterminate cases were subjected to an immunoblot confirmatory test (TESA-cruzi, Biomerieux Brazil). Positive cases were traced by an MSF physician, with the collaboration of MSF cultural mediators, who encouraged patients to collect their results and referred them to the CTD for clinical staging and treatment, after confirming availability on the hospital ward waiting list. If patients needed to cover their transport expenses, screening tests expenses were covered by MSF and CTD. Clinical staging and treatment, follow-up visits at CTD were free-of-charge for patients. Tracing of those who did not return to receive the results and those who were lost to follow up was done by cultural mediators through phone calls, Short Message Service (mobile SMS) and e-mails, as opportune. Patients who did not undergo to staging and treatment were termed as ‘pre-treatment loss to follow-up”.

#### Patient assessment, treatment eligibility and drug administration

All confirmed cases of CD were assessed for treatment eligibility at the CTD. Patients were clinically evaluated and staged for their disease after performing standard Electrocardiogram (ECG), cardiac ultrasonography, 24 hours-ECG if needed, chest X-ray and study of the digestive tract (following Rezende’s protocol) [19]. Those found with co-morbidities and advanced cardiac or digestive impairment were referred for management to specialists of Sacro Cuore Hospital.

Individuals aged nine months to 60 years were considered eligible for treatment. The exclusion criteria for treatment are shown in Box 1.

**Box 1. Exclusion criteria for Benznidazole treatment, Bergamo, Italy**

- Unable to attend follow up visits
- Did not sign informed consent. In the case of a minor, consent needs to be signed by the parents, or guardian.
- Contraindications to the Benznidazole treatment i.e. pregnancy, hepatic or kidney failure, history of alcohol or drug addiction.
- Severe or uncontrolled co-morbidities: Rheumatic pathologies, dermatopathy, neuropathies, malignancies, gastro-duodenal ulcer, previous severe cardiopathies, refractory hypertension, severe neurological disease
- Signs of chronic digestive tract disease e.g. intestinal obstruction, faecaloma, oesophageal dysfunction.
- Signs of severe chronic Chagas cardiopathy.
- Received a previous complete course of Chagas treatment.

The first-line medication was Benznidazole (Abarax, ELEA, Buenos Aires, Argentina) given at an oral dose of 5 mg/kg twice a day for 60 days. The second-line drug in case of intolerance was Nifurtimox (Lampit, Bayer, Germany) given at an oral dose of 10mg/kg/day for 60 days.

The first week of Benznidazole treatment was administered under straight medical control at CTD. Subsequently, patients were followed-up on an ambulatory basis: drugs were given for four weeks following which the patient was scheduled to return to CTD for a single follow up visit. The patient then completed treatment on an ambulatory basis for a further month. Side effects were monitored clinically and patients also underwent laboratory assessments of blood count, hepatic and renal functions at least once during the first month of treatment. Individuals not eligible for treatment and individuals having completed treatment were followed up regularly according to their clinical status with an ECG and a physical consultation.

#### Standardized management outcomes

For the purposes of this study, treatment data were standardized and included: treatment completed–completion of at least 60 days of treatment; lost to follow up–a patient who did not turn up for a scheduled appointment and could not be traced; death–a patient who died for any reason while on treatment; stopped–a patient whose treatment had to be stopped by the clinician due to drug related adverse events or because of patient refusal to continue treatment; alive and on treatment–a patient still on treatment at the end of the study period.

### Study population and study period

The study population included all PLAO screened for CD in Bergamo between 1^st^ June 2012 and 30^th^ June 2013. The study was conducted between 1^st^ July 2013 and 28^th^ February 2014.

### Data collection and analysis

Data related to the study objectives were entered from baseline patient information sheets and patient files into a dedicated password-protected electronic database. This database had information on screening, diagnosis and treatment and patient management outcomes were updated on a regular basis by attending clinicians. Data were cross-validated using patient information sheets and patient clinical files. Analysis was performed using EpiData Analysis software (version 2.2.2.182, EpiData Association, Odense, Denmark). Descriptive statistics including proportions, medians and means were used where relevant. Chi-square test or Fisher’s exact test and Kruskal-Wallis test were used to compare categorical and continuous variables respectively. A P value of <0.05 was considered significant.

### Ethics

Permission for the project was received from the Verona and Rovigo Provincies Health Authority Ethics Board, Verona, Italy, under the “Provvedimento n. 5 del 08/03/2013”. In addition this study met the Médecins Sans Frontières (Geneva, Switzerland) Ethics Review Board approved criteria for analysis of routinely-collected program data. All data analyzed were anonymized. Enrolled patients were followed up after the study period with ECG, *T*. *cruzi* serology and clinical assessment with the appropriate periodicity depending on the stage of the disease.

## Results

### Numbers screened and diagnosed with CD

A total of 1305 individuals were screened for Chagas of whom 223 (17%) were found positive. Screened individuals were mainly of Latin American origin and included 1176(90%) individuals born in Bolivia, 47(4%) Ecuador, 24(2%) in Peru, 13(1%) in Brazil, 11(0.8%) in Argentina, two (0.2%) in Chile, two (0.2%) in El Salvador, and one (0.1%) in Panama. Twenty six people (2%) were born in Italy but had risk factors of having CD and were also screened by the program. Country of origin was not available for two individuals (0.2%). The results were sent back to OIKOS after about two months from the date of blood specimen collection—a delay linked to the lack of dedicated laboratory staff within the project and to the distance between the laboratory and OIKOS.

**Fig 1:**
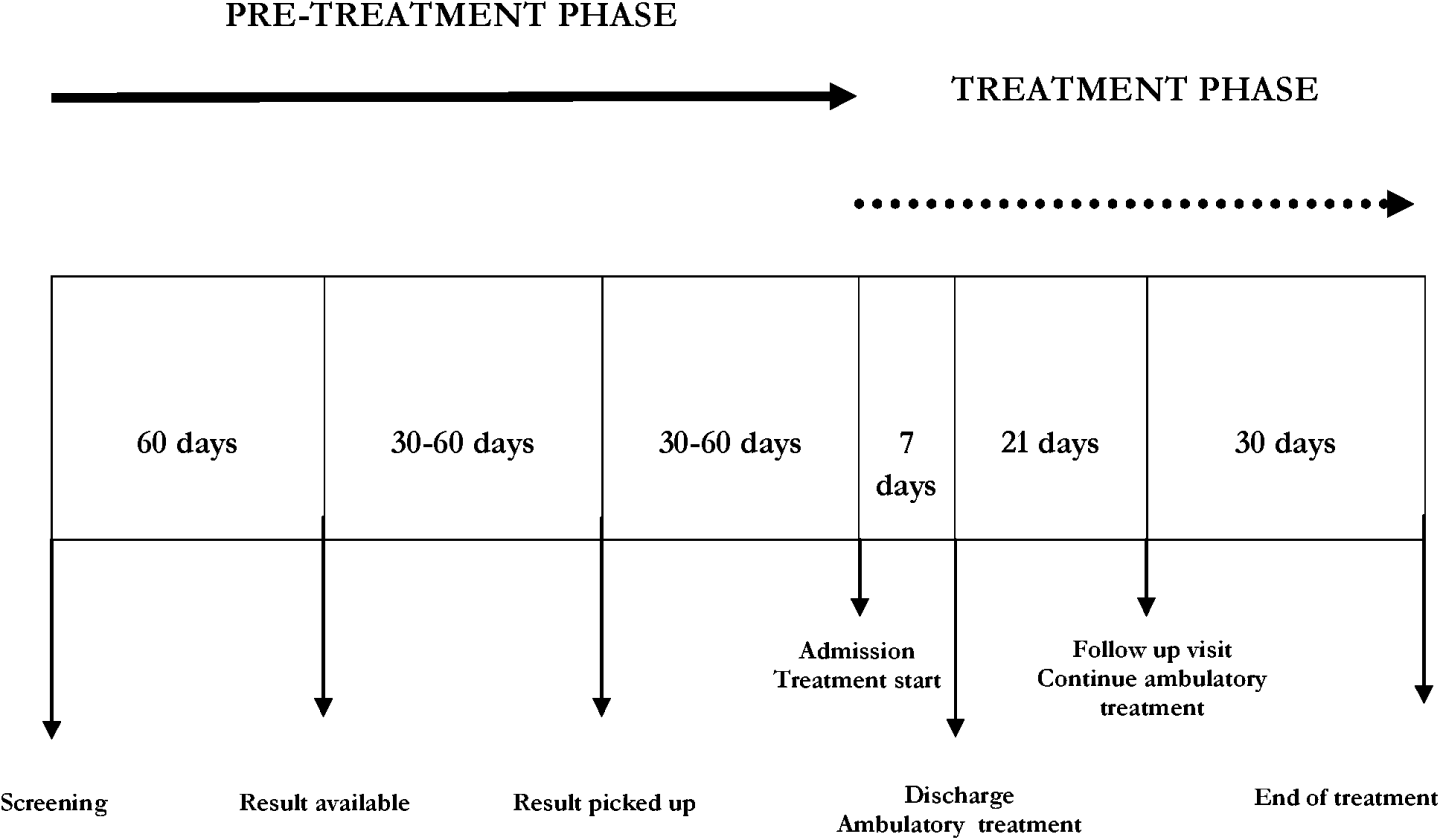
Timing of program components in a Chagas disease program in Bergamo, Italy, 1st June 2012-30th June 2013.

Fig 1 shows the journey and timing between screening, blood results becoming available and treatment initiation. Reasons of loss to follow-up were not investigated because out of the purpose of our observational study.

All positive individuals were of Bolivian origin including one child born in Italy from a mother of Bolivian origin. Serology was initially considered undetermined in 25 (2%) individuals. At the time of the study, confirmation testing was not available for undetermined cases. Table 1 shows the prevalence of CD stratified by demographic and social characteristics. For the statistical analysis two groups were compared to each other: those with confirmed CD and those without CD. The prevalence of CD was significantly higher among females, those aged 60 years and above, those who lived in a rural setting or mud/bamboo houses in their home country.

**Table 1:** Characteristics of people screened and diagnosed with Chagas disease, Bergamo, Italy (1^st^ June 2012-30^th^ June 2013).

Variables	Number screened	Confirmed with Chagas n (%)	P value
Gender			
Male	458	60 (13)	0.005
Female	847	163 (19)	
Age (years)			
<18	153	1 (<1)	<0.001
18–59	1119	208 (19)	
≥60	28	13 (46)	
Not recorded	5	1 (20)	
Home country prevalence of Chagas disease ^a^			
< 1% (Low)	15	0	<0.001
1–10% (Moderate)	37	0	
> 10% (High) ^b^	1253	223 (18)	
Home country setting			
Rural	632	132 (21)	<0.001
Urban	651	89 (14)	
Not recorded	22	2 (9)	
Type of home country housing			
Mud or bamboo material	747	164 (22)	<0.001
Other construction material	532	56 (11)	
Not recorded	26	3 (12)	

^a^ Reference [20]

^b^ includes children born in Italy from mothers of Latin American origin.

### Numbers eligible and initiated on treatment and time delay between diagnoses and treatment initiation

Of 223 individuals confirmed with CD and who should have received treatment, 13(5%) were excluded as they were aged 60 years or over. Of the remaining 210 individuals who were scheduled to receive treatment 102(49%) were lost to follow up in the pre-treatment phase and 108(51%) were initiated on treatment. The median delay from diagnosis to treatment was 4 months (Inter Quartile Range, IQR = 2.8–5.9 months; range 0.7–16.6 months).

Table 2 shows the characteristics of individuals lost to follow up in the pre-treatment phase. For the statistical analysis two groups were compared to each other: those who initiated on treatment and those who were lost to follow up before treatment initiation. Individuals who lived in mud/bamboo houses in their home country (*P* = 0.01had a significantly higher chance of being lost to follow up.

**Table 2:** Characteristics of individual lost to follow up (LTFU) in the pre treatment phase and of individuals initiated on treatment, Bergamo, Italy (1^st^ June 2012-30^th^ June 2013).

Variables	LTFU n (%)	Initiated on treatment n (%)	P value
Total	102	108	
Gender			
Male	26 (25)	31 (29)	0.6
Female	76 (75)	77 (71)	
Age (years)			
Median (range)	45 (4–59)	43 (17–60)	0.4
Home country setting			
Rural	63 (62)	61 (56)	0.3
Urban	37 (36)	47 (44)	
Not recorded	2 (2)	0	
Type of home country housing			
Mud or bamboo material	83 (81)	72 (67)	0.01
Other construction material	17 (17)	35 (32)	
Not recorded	2 (2)	1 (1)	
Time delay from diagnosis to blood result collection (months)			
Median (range)	2.1 (0.7–9.7)^a^	1.7 (0.7–17.4)^b^	0.5

^**a**^ Data available for 56 people

^b^ Data available for 79 people

### End of treatment management outcomes

Of 108 individuals who started Benznidazole treatment, 74(69%) were in the undetermined phase, and 34(31%) were in the chronic phase (14 with cardiac, 15 with digestive, four with mixed cardiac and digestive and one with neurological involvement).

Among 108 patients, 63(58%) completed treatment, 36(33%) stopped treatment, six (6%) were lost to follow up, and three (3%) were still on treatment at the time of study censoring. Of the 36 individuals who stopped treatment, 33 (92%) stopped due to drug related adverse events (under clinician judgement), one because of pregnancy, and two patients refused to continue treatment.

**Fig 2:**
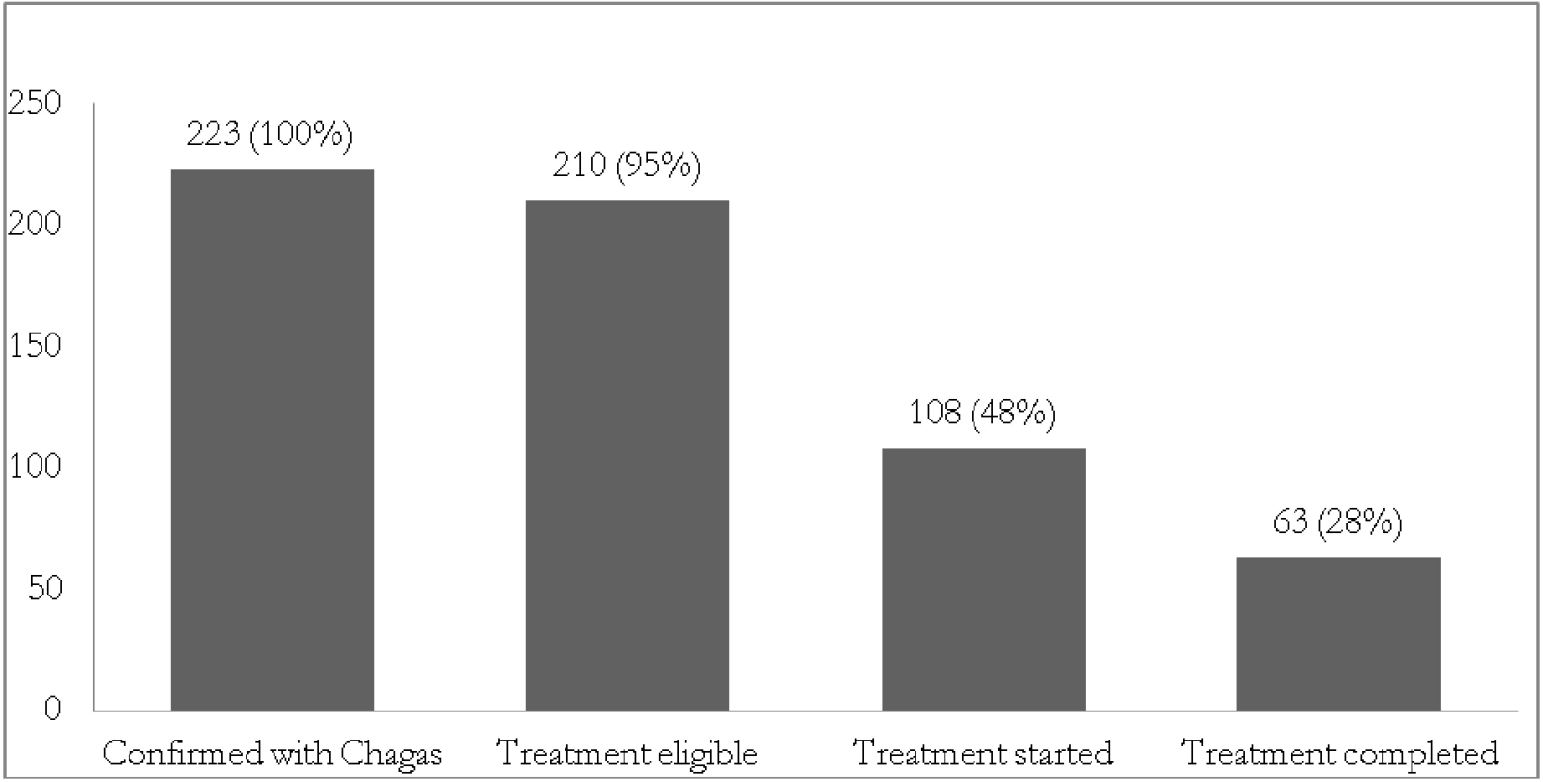
Treatment cascade (including diagnosis, treatment eligibility, treatment initiation, and treatment completion) of a Chagas disease program in Bergamo, Italy, 1st June 2012-30th June 2013.

Fig 2 shows numbers and proportion of drop-out from diagnosis and treatment completion.

### Drug related side effects

Among people who started treatment 59(55%) experienced at least one drug related adverse event. There were a total of 72 adverse event episodes: the most frequent were skin rash 47(66%) followed by headache 6(8%) and neuropathy 4(6%). In 27(58%) and 4(100%) episodes of skin rash and neuropathy, respectively, treatment was stopped by the clinician. Of 59 individuals who experienced adverse events, 33 (56%) stopped treatment. Two patients had leukopenia associated with skin rash and one patient had simple leukopenia: all of them had to stop treatment. No one stopped for insurgence of hepatitis. There were no reported deaths.

## Discussion

The Italian National Health System guarantees urgent and continuative care for chronic disease free-of-charge to all people of foreign origin living in Italy, regardless of whether they are documented or not. This regulation practically means that inpatient services are always provided free-of-charge, while outpatient services are provided free of charge depending on the type of disease. As yet, outpatients with CD do not benefit from access to services free-of-charge.

The studied model-of-care has been implemented by MSF and partners in order to fill the gap between the offer of the Italian National Health System for CD and the real access to health care in a high prevalence area in Italy. In fact, by the time of the study period, in Italy no national protocol for CD management was available and in the Bergamo area no free-of-charge service of voluntary universal screening was possible in the local public health facilities for the population at risk of the disease. In order to assure specific competence and give strong points of reference to the patients a partnership between well-known specialized institutions was established. To reduce time losses and to concentrate staging procedures and care, affected people were managed as in-patients for a short period (3–5 days) and follow-up was reduced to phone contact and one visit at one-month of treatment and one or two blood test controls. Initial treatment management as in-patients was deemed necessary because the nearest centre of reference was located far from Bergamo, 130 km away, making daily management as outpatients not feasible. This choice was a balance between correct clinical management and meeting the PLAO’s needs to avoid more job day losses.

Our study is the first assessing the process from screening to treatment initiation and completion among individuals screened at community level and diagnosed with CD. The study shows that about two in ten patients screened in our program had CD and all were of Bolivian origin. In our study, the prevalence of CD was higher in women, elderly people, and those who had lived in a rural setting before coming to Italy: while the last two findings are in line with previous studies the fact that a higher proportion of positive cases were seen among women needs to be interpreted with caution. In fact, a higher proportion of women adhered to the screening programme compared to men: this reflects both the effective gender distribution among PLAO living in Bergamo area [16],and the fact that women may have been more sensitive to the health promotion and education campaign. Furthermore, women have different job profiles compared to men, which may have allowed greater work-place flexibility and available time to seek health care.

Drop-outs along the cascade of care resulted in less than one third of individuals with CD completing treatment. Attrition from care was mainly due to pre-treatment losses-to-follow up and serious adverse drug reactions. With such low levels of treatment uptake and medication tolerance, at least following our mode-of-care and this individual experience, it is unlikely that the WHO roadmap for increasing access to treatment and boosting transmission prevention by 2020 would be achieved—even in Europe [9]. Ways forward to address these challenges are urgently needed.

The strengths of the study are that we used a partnership approach involving the Latin American community which may foster treatment advocacy in the future, data were validated using patient files, side effects were closely monitored and documented by clinicians and a focus was paced on PLAO who are most at risk (people of Bolivian origin, who comprised over 50% of the PLAO living in the study area). In addition, the findings come from for an operational setting and are likely to reflect the ground reality. This is also the first time that attrition along the entire cascade of care is being documented.

There are also some study limitations: first of all, the project was conducted grafting on an already on-going, less intensive program. The MSF intervention permitted to expand the screening sessions, empower health education and increase enormously awareness and access to diagnosis, but the “centralized” management of the care could have been engulfed by this intensive approach. Moreover, for the study purposes, efforts to trace patients by MSF health promoters and treat them were concentrated in a one-year period and patients not reached in this period were considered pre-treatment lost to follow-up: the process of tracing continued after the end of the study period and more patients had had effective access to care. Another study limitation is that we do not know the reasons for losses to follow up in the pre-treatment and treatment phases which may even include unascertained deaths and other factors such as migration back to birth country, fear from the disease or stigmatization, inability to take days off from work to attend medical consultations.

Our study has some implications: first, a big proportion (about half) of the diagnosed cohort was lost to follow-up prior to initiating treatment. We do not know the exact reasons for this result which would definitely merit further qualitative research. In our study 48% of patients with CD started treatment and 28% completed it. Only one study on CD management outcomes by Perez Ayala et al. [14] showed similar results regarding treatment completion (29%) and adverse events (52%), but there were less treatment stops (21%), less drug related treatment stops (30%) and less pre-treatment losses to follow up (45%). In their study, however, they did not focus on reasons of pre-treatment loss to follow up and their study period was seven years, thus offering more time to implement a tracing strategy for cases who did not show up after the screening results.

In order to explain the poor adherence after screening in our program we can try to address a number of challenges from the patient perspective. After being diagnosed, there is a considerable delay of four to six months before treatment can be received, a fact which is not considered to have clinical impact but can influence patient compliance. Disease staging, treatment and follow-up, although well organized, time-sparing and free-of-charge, were highly centralised in the reference Centre located far from Bergamo. The staging process and initial treatment period under medical control, although reduced at minimum in our mode-of-care, may imply loss of work time and related revenue. The finding that individuals who were lost to follow-up were likely to come from lower socio-economic backgrounds (i.e, lived in mud/bamboo houses in their home country) tends to support such thinking. In this regard, a way forward in improving access to care would be to decentralize diagnosis and treatment to Bergamo city, in order to permit a follow-up on an ambulatory basis, limiting loss of work time for the patients and less travel related expenses. [21] On the other side, we do recognize that for the Italian health system a structured outpatient service for CD is not provided yet, in general, both to Italians and people of foreign origin therefore all staging test costs would be at patient charge.

The introduction and use of rapid point-of-care diagnostic tests would have avoided the two-month delay required for receiving results from a central laboratory [22], although this may be saddled with a lower diagnostic accuracy: for this reason in our model-of-care WHO diagnostic gold standard with two Elisa essays was chosen.

Second, about half of all patients initiating treatment encountered side effects to Benznidazole and one third were compelled to stop treatment. Such high incidence of side effects has already been reported in the literature both in Latin America [23–25] and in Spain[11]. In case of side effects, the only alternative treatment is Nifurtimox which also has a similar if not worse toxicity profile. This highlights even more the urgent need for research and development of newer and safer drugs and the rapid testing of the molecules under development in phase 2 and phase 3 clinical trials [26].

Third, despite the encouraging finding that some patients completed treatment, with our limited study period we do not know if they were actually cured from the parasite. This is an important practical challenge as there is currently no rapid internationally validated laboratory test for confirming cure: some promising cure biomarkers are under research [27], however these results were not available during the study period. The inability to ascertain a definite cure, unless with a long serological and clinical follow-up, may also influence acceptability of this rather toxic treatment and also explain some of the losses to follow-up.

In conclusion, if we are in the need to accelerate the pace towards bridging the current 99% global access gap for CD treatment (including in Italy), it is urgent to increase awareness on this neglected disease at different levels, to adapt the model-of-care to the epidemiological scenario and make newer diagnostics and safer drugs available. This would be particularly challenging in a country, Italy, where CD has a relevant prevalence [28] with a patchy distribution. We appeal to the Italian Health Authorities to take the first steps in advocating for a change in the current paradigm.

## Supporting Information

S1 Checklist STROBE Checklist. (DOCX)
